# Descriptive Epidemiology of Human Thyroid Cancer: Experience From a Regional Registry and The “Volcanic Factor”

**DOI:** 10.3389/fendo.2013.00065

**Published:** 2013-06-04

**Authors:** Pasqualino Malandrino, Claudia Scollo, Ilenia Marturano, Marco Russo, Martina Tavarelli, Marco Attard, Pierina Richiusa, Maria Antonia Violi, Gabriella Dardanoni, Riccardo Vigneri, Gabriella Pellegriti

**Affiliations:** ^1^Endocrinology, Department of Clinical and Molecular Biomedicine, University of Catania, Garibaldi-Nesima Medical Center, Catania, Italy; ^2^Endocrinology, “Ospedali Riuniti Villa Sofia – Cervello” Hospital, Palermo, Italy; ^3^Endocrinology, Diabetology and Metabolism, Department of Internal and Specialistic Biomedicine, University of Palermo, Palermo, Italy; ^4^Endocrinology, Department of Clinical and Experimental Medicine, University of Messina, Messina, Italy; ^5^Sicilian Regional Epidemiology Observatory, Palermo, Italy

**Keywords:** thyroid cancer, epidemiology, registry, incidence, risk factors, papillary, volcanoes

## Abstract

Thyroid cancer (TC), the most common endocrine tumor, has steadily increased worldwide due to the increase of the papillary histotype. The reasons for this spread have not been established. In addition to more sensitive thyroid nodule screening, the effect of environmental factors cannot be excluded. Because high incidences of TC were found in volcanic areas (Hawaii and Iceland), a volcanic environment may play a role in the pathogenesis of TC. In January 2002, the Regional Register for TC was instituted in Sicily. With a population of approximately five million inhabitants with similar genetic and lifestyle features, the coexistence in Sicily of rural, urban, industrial, moderate-to-low iodine intake, and volcanic areas provides a conducive setting for assessing the environmental influences on the etiology of TC. In Sicily, between 2002 and 2004, 1,950 new cases of TC were identified, with an age-standardized rate (world) ASR(w) = 17.8/10^5^ in females and 3.7/10^5^ in males and a high female/male ratio (4.3:1.0). The incidence of TC was heterogeneous within Sicily. There were 2.3 times more cases in the Catania province (where most of the inhabitants live in the volcanic area of Mt. Etna): ASR(w) = 31.7/10^5^ in females and 6.4/10^5^ in males vs. 14.1 in females and 3.0 in males in the rest of Sicily. Multivariate analysis documented that residents in the volcanic area of Mt. Etna had a higher risk of TC, compared to the residents in urban, industrial, and iodine deficient areas of Sicily. An abnormally high concentration of several chemicals was found in the drinking water of the Mt. Etna aquifer, which provides water to most of the residents in the Catania province. Our data suggest that environmental carcinogen(s) of volcanic origin may promote papillary TC. Additional analyses, including cancer biological and molecular features, will allow a better understanding of risk factors and etiopathogenetic mechanisms.

## Introduction: Thyroid Cancer Epidemiology and Trend

Thyroid cancer (TC) is the most frequent endocrine neoplasm (Curado et al., 2007), which occurs two to four times more frequently in females than in males (Parkin et al., 2005). Its incidence has continuously increased worldwide in recent decades. This increase has occurred in many regions, with variable entity and independent of initial recorded levels, as indicated by data from population-based cancer registries, particularly in the Western world, such as the United States (Davies and Welch, 2006), Canada, and in many European Countries (Leenhardt et al., 2004; Yu et al., 2010) (Table 1).

**Table 1:** **Age-adjusted thyroid cancer incidence rates from different population-based cancer registries**.

Authors	Data source	Study period	No. of cases		Incidence rates*	
			Males	Females	Males	Females
Chen et al. (2009)	SEER	1988–2005	7,458	23,308	2.5–5.1	6.4–14.9
Burgess (2002)	ANCSCH	1982–1997	–	–	1.27–2.04	2.89–5.52
Scheiden et al. (2006)	MTR	1983–1999	74	236	3.0–5.7	9.0–10.5
Sassolas et al. (2009)	TCR-RA	1998–2006	1,256	4,111	2.47–3.95	8.35–12.70
Rego-Iraeta et al. (2009)	PRUHV	1978–2011	70	252	0.33–3.24	1.56–8.23
Netea-Maier et al. (2008)	NCR	1989–2003	1,451	3,629	0.9–1.1	3.1–3.1
AIRTUM Working Group (2006)	AIRTUM	1998–2002	675	2.579	5.2	15.5
Pellegriti et al. (2009)	SRRTC	2002–2004	366	1,584	3.7	17.8

*Range provides incidence at the beginning and end of the study period.

*SEER, Surveillance Epidemiology and End Results; ANCSCH, Australian National Cancer Statistics Clearing House; MTR, Morphologic Tumor Registry (Luxembourg); TCR-RA, Thyroid Cancer Registry of Rhône-Alpes region (France); PRUHV, Pathology Registry of the University Hospital of Vigo (Spain); NCR, Netherlands Cancer Registry; AIRTUM, Italian Association of Cancer Registry; SRRTC, Sicilian Regional Registry for Thyroid Cancer (Italy)*.

Based on data from 1999 through 2008, TC represents the fastest growing cancer, in terms of prevalence, in the United States among men and women of every racial and ethnic background (except American Indian or Alaska Native men) (Simard et al., 2012).

The epidemiological data of Surveillance Epidemiology and End Results (SEER) reported an annual incidence of TC of 5.9/10^5^ individuals in men and 17.3/10^5^ in women from 2005 through 2009 and an age-adjusted death rate of 0.5/10^5^ in both men and women per year (Howlader et al., 2012).

The reasons for the continuously increasing incidence of TC are unclear and controversial. The increase in TC might be attributed to more intensive and sensitive thyroid nodule diagnostic procedures (thyroid ultrasound and fine-needle aspiration biopsies) and the results of improved diagnosis of subclinical cancers (Davies and Welch, 2006; Kent et al., 2007). Methodologies for the pathological examination of the excised thyroid gland could also explain this phenomenon. Recently, pathologists have begun to dissect thinner slices of the gland (less than 2 mm) and frequently sample not only macroscopically evident lesions but also the entire thyroid gland (Pazaitou-Panayiotou et al., 2007; Boucek et al., 2009). The increased microcarcinoma prevalence and the rising number of incidentally discovered TCs are consistent with these hypotheses (Leenhardt et al., 2004; Davies and Welch, 2006). Recently, it has been hypothesized that environmental factors (or chemical agents) might explain this increasing incidence (Leenhardt and Grosclaude, 2011) and, consequently, that a more accurate screening cannot explain the (likely) multifactorial mechanisms that might account for this increase.

To the best of our knowledge, there are no available data in the recent literature, but we can hypothesize that the more diffuse use of sensitive diagnostic procedures has reached a plateau in many countries because of standardized diagnostic protocols. The incidence of TC has not been steady, as reported in the case of prostate cancer, according to data from the SEER registries (annual percentage change of −1.5 in the period 2000–2009) (Howlader et al., 2012).

The relevant increase of TC generally refers to papillary cancer less than 1 cm in diameter (microcarcinoma), which leads to an “epidemic of micropapillary TC” (How and Tabah, 2007). A recent article emphasized that the most common type of TC in patients older than 45 years is microcarcinoma (Hughes et al., 2011). Nevertheless, the incidence of TCs of all sizes (including the large ones, ≥4 cm in diameter) increased in the 1988–2005 period in the United States (Chen et al., 2009) as well as in other countries (Rego-Iraeta et al., 2009).

Furthermore, the increased incidence of TC is nearly exclusively due to the papillary histotype, with no significant change in the other histotypes. This finding suggests that specific carcinogens might favor the molecular abnormalities typical of papillary cancer.

Controversy has emerged regarding the differences in the incidence of TC in different ethnic groups. In addition to the possible effect of genetic factors, variable access to medical care has been hypothesized to be a contributing to this incidence. Although the incidence of TC is lower among Hispanics and Afro-Americans compared to Caucasians (Simard et al., 2012), very recent data report the greatest acceleration in incidence rate of papillary TC among black females (Aschebrook-Kilfoy et al., 2013).

At present, carcinogens that may trigger TC or contribute to its progression have not been established. The risk factors for TC include exposure to ionizing radiations (Nikiforov, 2006), a history of goiter or thyroid nodules or a family history of TC. In addition, dietary factors (Markaki et al., 2003), westernized lifestyle and obesity (Kitahara et al., 2011), or anthropogenic pollution (i.e., caused by human activities) cannot be excluded as factors. Concerning dietary factors, several studies have investigated the impact of iodine intake on the risk of TC onset. Whether adequate iodine consumption has a protective role against thyroid carcinogenesis has not been determined. These studies concur with the observation that the ratio of papillary to follicular TCs is higher in geographical areas with adequate iodine intake than in areas with moderate-to-severe iodine deficiency (Peterson et al., 2012).

Some of the highest incidences of TC were found in Hawaii (Goodman et al., 1988; Kolonel et al., 1990) and Iceland (Arnbjornsson et al., 1986), both volcanic areas. A comparison of the same ethnic groups living in Hawaii or in other geographical areas indicated that the incidence of TC was significantly higher in the first group, which suggests that environmental factors might play a role (Goodman et al., 1988).

Large population-based cancer registries are appropriate for studying the incidence of cancer and genetic factors in relation to the environment. These registries require a close cooperation between clinicians, epidemiologists, and environment specialists.

Creating such a registry was the aim of our research; the study began planning early in 2000 on the Mediterranean island of Sicily.

## The Sicilian Regional Registry for TC

Sicily, with approximately five million inhabitants, offers the opportunity to study different environmental factors that can influence the incidence of TC. Sicily has a homogeneous population in terms of genetics and lifestyle, with similar access to medical assistance and, as an island, is very well geographically delimited. In Sicily, there is the concomitant presence of rural and urban areas, industrial and non-industrial areas, and areas of low or adequate iodine intake. Sicily also hosts Mt. Etna, the highest and most active volcano in Europe.

Data collection was organized through the institution of the Sicilian Regional Registry for Thyroid Cancer (SRRTC). Patients were identified by a systemic survey (three to four times each year) of all Sicilian (public and private) morbid pathology services. Data were recorded in a computerized archive and were correlated with population data (Italian National Institute of Statistics, 2001) (Pellegriti et al., 2009).

We evaluated the age-standardized incidence rate for the world population (ASR_w_) in each of the nine provinces in Sicily in the 3-year period of 2002–2004. We found a more than twofold increased incidence of TC in the province of Catania (ASR_w_ *F* = 31.7/10^5^; *M* = 6.4/10^5^), where approximately 80% of the residents live in the volcanic area of Mt. Etna, in contrast to the rest of Sicily (ASR_w_ *F* = 14.1/10^5^; *M* = 3.0/10^5^) (Table 2). The incidence of TC was not different between the industrial and the non-industrial areas nor between the areas of sufficient and deficient iodine intake. A higher ASR_w_ was observed in urban areas compared to rural areas. A similar finding was reported by Sassolas et al. (2009), who documented in France a higher incidence of TC in the urban cantons of the Rhône-Alpes region than in the non-urban areas of the same region. A non-uniform presence of medical specialists between the urban and non-urban areas may explain this difference. Although a different access to medical assistance between the urban and rural areas is reasonable, this variable access is not likely to explain the difference between the Catania province and the other eight Sicilian provinces. The Catania province includes the Catania metropolitan area (approximately 650,000 inhabitants) and a large rural area with approximately 350,000 inhabitants. A similar urban/rural ratio is present for the provinces of Palermo and Messina, where the incidence of TC is much lower than in Catania. In Sicily, a similar access to medical assistance is indicated by the number of routine biochemical laboratory tests per inhabitant: 6.89, 7.42, and 6.18 in 2007 in the provinces of Catania, Messina, and Palermo, respectively (Sicilian Public Health Report 2008, Appendix 1, www.gurs.regione.sicilia.it). These three provinces include the largest Sicilian cities, approximately 60% of the Sicilian population, as well as the largest medical schools and major hospitals. Moreover, the Poisson regression analysis adjusted for all environmental characteristic confirmed that, compared with residents of urban, industrial, and iodine deficient areas, living in the volcanic area of the province of Catania was an independent risk factor of TC in both females (odds ratio = 2.242, 95% C.I. = 2.020–2.488) and males (odds ratio = 2.052, 95% C.I. = 1.652–2.565) inhabitants (Table 3).

**Table 2:** **Thyroid cancer incidence in Sicily: age-standardized rates for the world population (ASR_w_) for the entire island and by environmental characteristic**.

Environmental characteristic	No. of residents	Females			Males			p*
		no. of cases	ASR_w_	(95% C.I.)	no. of cases	ASR_w_	(95% C.I.)
Total for all Sicily	4,980,352	1584	17.8	16.9–18.7	366	3.7	3.3–4.1	
Volcanic environment
Yes (Catania Province)	1,059,811	599	31.7	29.1–34.3	130	6.4	5.2–7.5	<0.001
No (Sicily without Catania Province)	3,920,541	985	14.1	13.2–15.0	236	3.0	2.6–3.4	
Rural	1,133,529	332	16.4	14.6–18.2	73	3.3	2.5–4.3	0.003
Urban	3,846,823	1252	18.2	17.2–19.2	293	3.9	3.4–4.3	
Industrial	364,110	93	14.1	11.2–17.0	26	3.8	2.2–5.4	0.005^†^
Non-industrial	4,616,242	1491	18.1	17.2–19.0	340	3.7	3.3–4.1	
Iodine deficiency	208,512	68	19.2	14.4–23.8	18	4.4	2.2–6.6	0.0084
Iodine sufficiency	4,771,840	1516	17.7	16.8–18.4	348	3.7	3.3–4.1	

*Poisson regression adjusted for sex and age.

*^†^When excluding volcanic area (where no industrial zone is present) *p* = 0.46*.

**Table 3:** **Thyroid cancer incidence in Sicily: odds ratio by environmental characteristic**.

Environmental characteristic	No. of residents	Females			Males			p*
		no. of cases	OR	(95% C.I.)	no. of cases	OR	(95% C.I.)
Volcanic environment
Yes (Catania Province)	1,059,811	599	2.242	2.020–2.488	130	2.052	1.652–2.565	<0.001
No (Sicily without Catania Province)	3,920,541	985	1		236	1		
Urban	3,846,823	1252	1.056	0.929–1.200	293	1.118	0.852–1.465	N.S.
Rural	1,133,529	332	1		73	1		
Industrial	364,110	93	0.998	0.805–1.237	26	1.174	0.773–1.784	N.S.
Non-Industrial	4,616,242	1491	1		340	1		
Iodine deficiency	208,512	68	1.163	0.900–1.502	18	1.468	0.897–2.401	N.S.
Iodine sufficiency	4,771,840	1516	1		348	1		

*Poisson regression adjusted for all environmental characteristic.

The mean age of the patients at the time of diagnosis and the female/male ratio were similar between the Catania province and the other Sicilian provinces. Most of TCs in the Catania province were papillary (94%), whereas in Sicily, excluding the Catania province, the percentage of papillary TC was lower (86%) (*p* < 0.0001). Although the mean tumor size at the time of diagnosis was slightly but significantly higher in Sicily, excluding Catania, with respect to the Catania province (1.50 ± 0.04 vs. 1.30 ± 0.05 cm; *p* = 0.002), the occurrence of both extrathyroid extension and lymph node metastases and the stage classification of tumors were not different between the two areas. Curiously, a higher percentage of multifocal TC was observed in the Catania province; i.e., 32 vs. 25% in the rest of Sicily (*p* = 0.001).

## Volcanic Activity: A Possible Risk Factor for TC

The presence of a high incidence of TC in volcanic areas in different parts of the world suggests that factors are present in the volcanic environment that may act as endocrine disruptors and carcinogens.

Several studies analyzed the incidence of TC among people living in the volcanic areas of Iceland (Hrafnkelsson et al., 1989), Hawaii (Kolonel et al., 1990), New Caledonia (Truong et al., 2007), and French Polynesia (Curado et al., 2007) and documented some of the highest occurrence rates reported in the literature. In French Polynesians, for example, the ASR_w_ are 5.4/10^5^ for males and 37.4/10^5^ for females. How the volcanic environment may influence thyroid carcinogenesis is not known, but non-anthropogenic carcinogens of volcanic origin may be responsible (Kung et al., 1981; Duntas and Doumas, 2009). The geologic processes of volcanism produce various elements and metals in abnormal concentrations in the soil, water, and atmosphere. Mt. Etna continuously delivers, and has for many decades, suspended particulate matter and gases: sulfur dioxide, hydrogen chloride, hydrogen fluoride, hydrogen sulfide, hydrochloric acid, sulfuric acid, ammonium sulfate, helium, and radon. These substances were detected in various volcanic eruptions (Hansell and Oppenheimer, 2004). Many of these potentially toxic compounds may become concentrated the environment (Kusky, 2008) and contaminate cultivated fields, including cultured vegetables and the animal food chain (Hogan and Burstein, 2007).

Several studies reported that various elements and chemicals (including HCO_3_, SO_4_, calcium, selenium, fluoride, chloride, magnesium, boron, manganese, iron, and vanadium and their salts and 222 radon) are often increased in water samples from various sources of Mt. Etna’s volcanic aquifer (Giammanco et al., 1996; Brusca et al., 2001; Aiuppa et al., 2002; D’Alessandro and Vita, 2003). Similar findings were reported in studies of water samples from other volcanic areas (Tilling and Jones, 1996; Martin-Del Pozzo et al., 2002). Mt. Etna is a large basaltic volcano with fissured and highly permeable lava layers interbedded with discontinuation layers of scarcely permeable pyroclastics. The main aquifers of Mt. Etna (overall calculated as 1.7 million cubic meters of water) lie between the volcanic rocks and the underlying impermeable sediments. A magmatic-type interaction occurs between the water and the volcanic soil; an enormous amount of CO_2_ produced by the volcanic degasification leads to the acidification of water and chemicals leaching from the basalt rock, especially in the lower south-southwestern and eastern flanks of the volcano (Brusca et al., 2001). Vanadium, for instance, is classified by the International Agency for Research on Cancer (IARC) as a possible human carcinogen (Group 2B) (IARC, 2006), which may influence thyroid function and cell proliferation. Experiments on rats documented the role of vanadium in affecting iodine metabolism and thyroid function by decreasing the thyroid peroxidase activity (Uthus and Nielsen, 1990). Vanadium may have a mitogen effect by stimulating the action of an unknown growth factor (Zhang et al., 2001; Ingram et al., 2003). Approximately 700,000 residents in the province of Catania receive water from the volcanic aquifer, and large agricultural areas are irrigated with water originating from the Mt. Etna aquifer. The role of water can explain why the increased cancer incidence is not related to the distance from the top of Mt. Etna. Messina (ASR_w_ *F* = 16.2/10^5^; *M* = 4.2/10^5^) and Enna (ASR_w_ *F* = 15.9/10^5^; *M* = 2.8/10^5^), which are adjacent to the Catania province, despite their proximity to Mt. Etna, do not have an increased incidence of TC. Messina and Enna provinces, however, receive most of their water from different aquifers (Peloritani Mountains for Messina and Erei Mountains for Enna) (Pellegriti et al., 2009).

However, the role of the different aquifer systems of the eastern Sicilian provinces has not been confirmed. The role of volcanic water as the vehicle for carcinogenic trace elements in initiating and promoting TC requires additional research.

The population’s exposure to one or more carcinogens of volcanic origin can occur in other ways, such as atmosphere, soil, or contaminated foods. The preliminary data indicate that the resident population is exposed to an abnormal concentration of trace elements that are increased in biological fluids. Mt. Etna (and other volcanoes) is a large emitter of trace elements; metals, separated from magma during degassing, are transported by rising gases, and as they approach the surface, they condense to small particles that are dispersed throughout the atmosphere. Because of the constant degassing and recurrent lava flow eruptions, atmospheric emissions of particulate Cd, Hg, Se, Cu, and Zn by Mt. Etna are equivalent to the amount of these elements released in the Mediterranean area by all of the anthropogenic activities (Buat-Ménard and Arnold, 1978). Most common winds in the Mt. Etna region blow from the north-northwest (Favalli et al., 2004). The Mt. Etna plume, therefore, moves mainly south-east, and Catania province inhabitants mostly live in the downwind areas of the ash fallout. Therefore, these individuals are exposed to high levels of these particulates. Moreover, vegetables and plants may accumulate various trace elements dispersed in the atmosphere. Several studies documented an increased amount of heavy metals in plants grown in volcanic areas. For instance, a significantly enhanced Hg content was found in *Pinus* of the Mt. Etna area (Barghiani et al., 1987). Other researchers found high levels of different trace elements (As, Sb, Bi, Cd, Cr, Pb, Zn) in vegetables grown in areas characterized by volcanic activity (Queirolo et al., 2000; Jung et al., 2002; Abiye et al., 2011). Vegetable contamination may occur not only through the atmospheric pollution but also by the presence of heavy metals in the irrigation water (Dahal et al., 2008).

Finally, environmental carcinogens of volcanic origin could be responsible for gene mutations favoring the thyroid carcinogenesis. In a recent paper, our group reported a higher rate of BRAF^(V600E)^ in eastern Sicily (hosting the Mt. Etna), compared to western Sicily (Frasca et al., 2008).

Table 4 provides a summary of the principal studies quoted in the present manuscript.

**Table 4:** **Summary of the principal studies quoted in the manuscript**.

Topic of the study	Authors	Years of the study	Geographical area	Main conclusions
Trace element concentration in water of volcanic aquifers	Tilling and Jones (1996)	1973–1991	Mt. Kilauea (Hawaii)	The overall chemistry of the volcanic aquifer is largely due to a hydrolysis reactions leading to the leaching of the rocks and is dependent on the volcanic degassing of CO_2_
	Martin-Del Pozzo et al. (2002)	1994–2000	Mt. Popocatepetl (Mexico)	Spring water content of ${\text{SO}}_{4}^{2-}$, CI^−^, F^−^, ${\text{HCO}}_{3}^{-}$, B, ${\text{SO}}_{4}^{2-}$ / CI^−^, Na^+^, Ca^2+^, SiO_2_, and Mg^2+^ measured before and during eruptions, show response to volcanic activity
	Giammanco et al. (1996)	1994–1997	Mt. Etna (Sicily)	Concentration of trace elements in groundwaters of Mt. Etna is temporally variable, linked to the volcanic activity
	Aiuppa et al. (2002)	1997–1999	Mt. Etna (Sicily)	Concentrations of B, V, and Mg in groundwaters of Mt. Etna exceed the maximum admissible limits
Trace element concentration in vegetables grown in volcanic areas	Barghiani et al. (1987)		Mt. Etna (Sicily)	Hg content is high in *Pinus* of the Mt. Etna area
	Queirolo et al. (2000)		Northern Chile	Concentration of As, Pb, and Cd are high in locally cultivated vegetables
	Abiye et al. (2011)		Ethiopia	Geogenic sources of Cd, Cr, Pb, and Zn are responsible for the high concentration of these metals in locally grown vegetables
	Dahal et al. (2008)		Nepal	The arsenic content in soil and plants is influenced by the degree of arsenic amount in irrigation water
Thyroid cancer incidence in volcanic areas	Goodman et al. (1988)	1960–1984	Hawaii	*M* = 3.1/10^5^; *F* = 8.1/10^5^
	Hrafnkelsson et al. (1989)	1955–1984	Iceland	*M* = 3.4/10^5^; *F* = 9.5/10^5^
	Truong et al. (2007)	1985–1999	New Caledonia	*M* = 10.4/10^5^; *F* = 71.4/10^5^
	Curado et al. (2007)	1998–2002	French Polynesia	*M* = 5.4/10^5^; *F* = 37.4/10^5^
	Pellegriti et al. (2009)	2002–2004	Mt. Etna (Sicily)	*M* = 6.4/10^5^; *F* = 31.7/10^5^
	Biondi et al. (2012)	2000–2009	Mt. Vesuvius (Campania)	PTC incidence crude rate = 9.0/10^5^ vs. 6.2/10^5^, respectively, in volcanic and non-volcanic area

## Conclusion

The rising number and the diffusion of sensitive diagnostic procedures may justify only part of the continuous increase of TC incidence. Additional studies are necessary to investigate the role of environmental factors, which may include the volcanic environment. Population-based cancer registries may allow a better understanding of the different agents and mechanisms underlying this increase.

There is a strong association between very high TC incidence and patient residence in volcanic areas. We propose the following considerations: (1) TC registries should be instituted in all volcanic areas with elevated population density because those populations are most likely at high risk for developing TC. However, not all volcanoes are necessarily the same in terms of environmental factors and vehicles for human exposure to carcinogens. (2) A primary aim of the research in this field should be to identify environmental factors and thyroid carcinogens in volcanic areas. These studies will enable us to understand the mechanisms and molecular alterations that might lead to TC. We can then develop specific interventions to prevent the increased risk of TC. (3) It is important to ascertain whether, in addition to TC, other cancers are favored by the volcanic environment. In the Mt. Etna area, an increased incidence of mesothelioma has been reported in the Biancavilla municipality district (Comba et al., 2003). Mesothelioma has been attributed to the increased inhalation of asbestiform fibers, a consequence of the generalized use of building materials from a local quarry. In Africa, endemic Kaposi’s sarcoma has been observed in areas containing volcanic clay minerals (Ziegler, 1993).

More than 500 million people in the world live in volcanic areas. Identifying the potential carcinogens involved in the pathogenesis of TC might help to promote preventive measures and understand the worldwide increase of this disease.

## Conflict of Interest Statement

The authors declare that the research was conducted in the absence of any commercial or financial relationships that could be construed as a potential conflict of interest.
